# Mortality After Postcolonoscopy Colorectal Cancer in the Veterans Affairs Health Care System

**DOI:** 10.1001/jamanetworkopen.2023.6693

**Published:** 2023-04-06

**Authors:** Charles J. Kahi, Laura J. Myers, Patrick O. Monahan, Barry C. Barker, Timothy E. Stump, Thomas F. Imperiale

**Affiliations:** 1 Department of Medicine, Richard L. Roudebush Veterans Affairs (VA) Medical Center, Indianapolis, Indiana; 2 Department of Health Services Research & Development, Richard L. Roudebush VA Medical Center, Indianapolis, Indiana; 3 Indiana University School of Medicine, Indianapolis; 4 The Regenstrief Institute, Indianapolis, Indiana; 5 Department of Biostatistics and Health Data Science, Indiana University School of Medicine, Indianapolis

## Abstract

**Importance:**

Postcolonoscopy colorectal cancer (PCCRC) refers to colorectal cancer (CRC) diagnosed after a colonoscopy in which no cancer was found and is reflective of colonoscopy quality at the individual and system levels. Colonoscopy is widely performed in the Veterans Affairs (VA) health care system, but the prevalence of PCCRC and its associated mortality are unknown.

**Objective:**

To examine PCCRC prevalence and its all-cause mortality (ACM) and CRC-specific mortality (CSM) within the VA health care system.

**Design, Setting, and Participants:**

This retrospective cohort study used VA-Medicare administrative data to identify 29 877 veterans aged 50 to 85 years with newly diagnosed CRC between January 1, 2003, and December 31, 2013. Patients whose colonoscopy occurred less than 6 months before CRC diagnosis with no other colonoscopy within the previous 36 months were categorized as having detected CRC (DCRC). Those who had a colonoscopy that did not detect CRC between 6 and 36 months before CRC diagnosis were categorized as having postcolonoscopy CRC (PCCRC-3y). A third group included patients with CRC and no colonoscopy within the prior 36 months. The final analysis of the data was performed in September 2022.

**Exposures:**

Prior receipt of colonoscopy.

**Main Outcomes and Measures:**

Cox proportional hazards regression (with censoring, last follow-up December 31, 2018) analyses were conducted to compare PCCRC-3y and DCRC for 5-year ACM and CSM after CRC diagnosis.

**Results:**

Of 29 877 patients with CRC (median [IQR] age, 67 [60-75] years; 29 353 [98%] male; 5284 [18%] Black, 23 971 [80%] White, and 622 [2%] other), 1785 (6%) were classified as having PCCRC-3y and 21 811 (73%) as having DCRC. The 5-year ACM rates were 46% vs 42% for patients with PCCRC-3y vs patients with DCRC. The 5-year CSM rates were 26% vs 25% for patients with PCCRC-3y vs patients with DCRC. In multivariable Cox proportional hazards regression analysis, there was no significant difference in ACM and CSM between patients with PCCRC-3y (adjusted hazard ratio [aHR], 1.04; 95% CI, 0.98-1.11; *P* = .18) and patients with DCRC (aHR, 1.04; 95% CI, 0.95-1.13; *P* = .42). However, compared with patients with DCRC, patients with no prior colonoscopy had significantly higher ACM (aHR, 1.76; 95% CI, 1.70-1.82; *P* < .001) and CSM (aHR, 2.22; 95% CI, 2.12-2.32; *P* < .001). Compared with patients with DCRC, patients with PCCRC-3y had significantly lower odds of having undergone colonoscopy performed by a gastroenterologist (odds ratio, 0.48; 95% CI, 0.43-0.53; *P* < .001).

**Conclusions and Relevance:**

This study found that PCCRC-3y constituted 6% of CRCs in the VA system, which is similar to other settings. Compared with patients with CRC detected by colonoscopy, those with PCCRC-3y have comparable ACM and CSM.

## Key Points

**Question:**

What are the prevalence and mortality associated with postcolonoscopy colorectal cancer (PCCRC) in the Veterans Affairs (VA) health care system?

**Findings:**

In this cohort study of 29 877 veterans with colorectal cancer (CRC), the prevalence of CRC diagnosed between 6 and 36 months of a colonoscopy that did not detect CRC was 6%. There was no significant difference in 5-year all-cause and cancer-specific mortality between patients with PCCRC and those with cancer detected by colonoscopy.

**Meaning:**

These findings suggest that colonoscopy quality in the VA system is comparable with that in other settings, but additional study of the factors associated with PCCRC and interventions to lower its occurrence is needed.

## Teaser

This cohort study examines all-cause and cancer-specific mortality among patients in the Veterans Affairs health system whose colorectal cancer was not detected by colonoscopy.

## Introduction

Colonoscopy is the centerpiece of colorectal cancer (CRC) prevention, both as a primary screening modality and as the final common pathway for other tests, such as sigmoidoscopy and stool tests for blood and/or DNA mutations.^1^ However, several studies^2,3,4^ have shown that colonoscopy affords imperfect protection against CRC, and some patients can be diagnosed with CRC after a colonoscopy that did not diagnose cancer but prior to the next guideline-recommended examination. These postcolonoscopy CRCs (PCCRCs) reflect the quality of the index colonoscopy and are considered a barometer of colonoscopy quality at the institutional and health care system levels. Most PCCRCs are attributed to missed or incompletely resected colorectal lesions (CRC or precancerous polyps), whereas fast-growing tumors account for a small proportion of PCCRCs. The observation that the variability in effectiveness of colonoscopy may be attributed to operator-dependent factors is critical because these factors are potentially remediable with educational and quality improvement initiatives.

Several studies^2,5,6,7,8,9,10,11,12,13,14^ have described PCCRC prevalence, clinical features, and risks factors and compared PCCRC characteristics with those of CRC detected at colonoscopy. Most studies report prevalence rates that range from approximately 2% to 10% of all CRC cases and a predilection for the proximal colon. However, few studies^7,9,11,12,13^ have assessed the outcomes of patients after their diagnosis with PCCRC, and some have reported conflicting findings regarding survival after diagnosis.

The Veterans Health Administration (VHA) system is one of the largest integrated health care systems in the US. Colorectal cancer screening is widely practiced and is guided by directives that emphasize the importance of high-quality screening and require measurement and reporting of colonoscopy quality metrics.^15^ Measuring and monitoring the rates of PCCRC and its associated factors and consequences for patient outcomes is critical to meaningful quality improvement and ensuring that veterans receive high-quality colonoscopies. In addition, studying PCCRC in the VHA has broad relevance, which extends beyond the confines of a single health care system. Given its size and the fact that the VHA serves as a reference standard in the health care field, the knowledge derived from a VHA-based study can help inform patients, practitioners, and policy makers in other health care organizations and enrich the CRC research field as a whole. We conducted a retrospective cohort study using national Veterans Affairs (VA)–Centers for Medicare and Medicaid Services (CMS) administrative data to determine PCCRC prevalence and its effect on all-cause mortality (ACM) and CRC-specific mortality (CSM).

## Methods

The study was approved as exempt research by the institutional review boards at Indiana University–Purdue University Indianapolis and by the research and development committees of the Richard L. Roudebush Veterans Affairs Medical Center in Indianapolis, Indiana. The institutional review boards approved a waiver of informed consent given the retrospective cohort design. The study followed the Strengthening the Reporting of Observational Studies in Epidemiology (STROBE) reporting guideline.

### Study Population and Definitions

This retrospective cohort study used VA-Medicare administrative data to identify all veterans aged 50 to 85 years with newly diagnosed CRC between January 1, 2003, and December 31, 2013. Patients with a history of CRC or surgical resection of CRC before January 1, 2003, were excluded, along with patients with a history of inflammatory bowel disease (Crohn disease or ulcerative colitis) or familial polyposis. Colorectal cancer was categorized as right-sided (cecum, ascending colon, hepatic flexure, or transverse colon) or left-sided (splenic flexure, descending colon, sigmoid colon, or rectum). Cancers were staged based on the 8th edition of the *AJCC Cancer Staging Manual*^16^; we excluded 395 patients with stage 0 disease (carcinoma in situ) because these patients are not considered to have invasive cancer.

We examined prior exposure to colonoscopy for any indication extending back to 1997 and defined 3 categories. Patients whose colonoscopy occurred less than 6 months before CRC diagnosis with no other colonoscopy within the previous 36 months were categorized as detected CRC (DCRC). Those who had a colonoscopy that did not detect CRC between 6 and 36 months before CRC diagnosis were categorized as having postcolonoscopy CRC (PCCRC-3y) based on World Endoscopy Organization (WEO) nomenclature. Patients with CRC and no prior colonoscopy within 36 months before CRC diagnosis were categorized as having no colonoscopy. In this group, 446 (7%) had a colonoscopy 3 to 10 years before CRC diagnosis, 4039 (64%) had a colonoscopy at more than 10 years (and not another one before the CRC diagnosis), and 1796 (29%) never had a colonoscopy. For all patients, we calculated a Charlson Comorbidity Index score based on 1 inpatient code or 2 or more outpatient codes separated by at least 30 days within the year before CRC diagnosis. We determined use of aspirin, nonsteroidal anti-inflammatory drugs, and statins. Race and ethnicity data were assessed by self-report (as Asian, Black, White, other, or unknown race and Hispanic or non-Hispanic ethnicity) and were included in the analysis because they are associated with differences in CRC outcomes.

### Data Sources

Data for this study were obtained from several electronic sources, including the Corporate Data Warehouse, VA-Medicare merged data, VA Vital Status File, and the VA/Department of Defense Mortality Data Repository. Detailed information regarding these electronic databases is outlined in the eMethods in Supplement 1.

### Statistical Analysis

The final analysis of the data was performed in September 2022. Descriptive statistics were used to characterize the demographic and clinical features of the study population, and we used the χ^2^ test or the 2-sided Fisher exact test to compare proportions and the unpaired, 2-tailed *t* test to compare continuous variables. The primary outcomes were ACM and CSM; both were analyzed using Cox proportional hazards regression to model the time to death after the diagnosis of CRC, adjusted for covariates. Patients were censored at the time of death or December 31, 2018 (the last date for which cause of death data were available). We reported unadjusted hazard ratios (HRs) and adjusted hazard ratios (aHRs) with 95% CIs, and the primary independent variable of interest was group membership (PCCRC-3y vs DCRC and no colonoscopy vs DCRC). We first performed univariate time-to-death analysis on each covariate using a Cox proportional hazards regression model to provide consistency between the univariate and multivariable analyses. Covariates with a 2-sided *P* ≤ .15 were included in multivariable models. The primary analysis excluded cancer stage because stage was considered a characteristic of cancer progression rather than a risk factor of group membership. However, the multivariable analysis was repeated to determine whether including cancer stage or excluding anatomical location of the cancer changed the results. In addition, a multivariable analysis was performed without the no colonoscopy group to include the index colonoscopy provider type as a covariate. We compared PCCRC and DCRC with regard to stage distribution, cancer location, and emergency presentation (obstruction, perforation, and peritonitis). A secondary analysis was conducted based on CRC diagnosed between 6 and 60 months of a colonoscopy that did not detect CRC (PCCRC-5y), with the cutoff point for colonoscopy set at 60 months before CRC diagnosis. R software, version 4.2.1 (R Foundation for Statistical Computing) was used for all analyses.

## Results

A total of 29 877 patients with CRC were identified (median [IQR] age, 67 [60-75] years; 29 353 [98%] male; 5284 [18%] Black, 23 971 [80%] White, and 622 [2%] other), with 21 811 (73%) classified as having DCRC and 1785 (6%) as having PCCRC-3y. Demographic and clinical features are summarized in Table 1. Colorectal cancer stage distribution at diagnosis was comparable among the 3 groups, except for a higher proportion of stage IV cancer among patients in the no colonoscopy group.

**Table 1.** Baseline Characteristics of the Study Patients^a^

Characteristic	Overall (N = 29 877)	DCRC (n = 21 811)	PCCRC (n = 1785)	No colonoscopy (n = 6281)	*P* value^b^
Age, y					
Mean (SD)	67.4 (9.0)	67.5 (9.0)	69.4 (8.3)	66.4 (9.1)	<.001
Median (IQR) [range]	66.0 (60.0-75.0) [50.0-85.0]	66.0 (61.0-75.0) [50.0-85.0]	69.0 (63.0-76.0) [50.0-85.0]	65.0 (60.0-74.0) [50.0-85.0]	
Sex					
Male	29 353 (98)	21 422 (98)	1756 (98)	6175 (98)	.80
Female	524 (2)	389 (2)	29 (2)	106 (1)	
Race					
Black	5284 (18)	3797 (17)	269 (15)	1218 (19)	<.001
White	23 971 (80)	17 557 (80)	1488 (83)	4926 (78)	
Other^c^	622 (2)	457 (2)	28 (2)	137 (2)	
Hispanic ethnicity					
No	27 926 (93)	20 458 (94)	1679 (94)	5789 (92)	<.001
Yes	1951 (7)	1353 (6)	106 (6)	492 (8)	
Statin use					
No	19 091 (64)	13 729 (63)	844 (47)	4518 (72)	<.001
Yes	10 785 (36)	8081 (37)	941 (53)	1763 (28)	
Missing	1	1	0	0	
NSAID use					
No	26 407 (88)	19 216 (88)	1530 (86)	5661 (90)	<.001
Yes	3469 (12)	2594 (12)	255 (14)	620 (9.9)	
Aspirin use					
No	22 784 (76)	16 530 (76)	1154 (65)	5100 (81)	<.001
Yes	7092 (24)	5280 (24)	631 (35)	1181 (19)	
CRC location					
Left or rectum	17 451 (58)	12 991 (60)	692 (39)	3768 (60)	<.001
Right	10 979 (37)	7958 (36)	999 (56)	2022 (32)	
Both	423 (1)	343 (2)	17 (1)	63 (1)	
Undefined	1024 (3)	519 (2)	77 (4)	428 (7)	
Family history of CRC					
No	29 336 (98)	21 366 (98)	1743 (98)	6227 (99)	<.001
Yes	540 (2)	444 (2)	42 (2)	54 (1)	
Current smoker					
No	18 047 (60)	13 378 (61)	1142 (64)	3527 (56)	<.001
Yes	11 829 (40)	8432 (39)	643 (36)	2754 (44)	
Weighted Charlson Comorbidity Index score					
Mean (SD)	2.32 (2.73)	2.26 (2.60)	2.85 (2.87)	2.35 (3.06)	<.001
Median (IQR) [range]	2.00 (0.00-3.00) [0.00-18.00]	2.00 (0.00-3.00) [0.00-18.00]	2.00 (1.00,4.00) [0.00-17.00]	1.00 (0.00-3.00) [0.00-17.00]	
Specialty					
Gastroenterology	16 954 (57)	15 944 (73)	1010 (57)	NA	<.001
Surgery or internal medicine	3152 (11)	2828 (13)	324 (18)	NA	
Other or unknown	9771 (33)	3039 (14)	451 (25)	NA	
Cancer stage					
I	9578 (32)	7904 (36)	663 (37)	1011 (16)	<.001
II	7518 (25)	5712 (26)	426 (24)	1380 (22)	
III	6682 (22)	4940 (23)	386 (22)	1356 (22)	
IV	6099 (20)	3255 (15)	310 (17)	2534 (40)	
Cancer stage group					
Early (I and II)	17 096 (57)	13 616 (62)	1089 (61)	2391 (38)	<.001
Late (III and IV)	12 781 (43)	8195 (38)	696 (39)	3890 (62)	
Index colonoscopy location					
VA	21 805 (92)	20 311 (93)	1494 (84)	NA	<.001
Non-VA	1791 (8)	1500 (7)	291 (16)	NA	
Died of CRC within 5 y of CRC diagnosis date					
No	21 255 (71)	16 462 (75)	1326 (74)	3467 (55)	<.001
Yes	8622 (29)	5349 (25)	459 (26)	2814 (45)	
Died of any cause within 5 y of CRC diagnosis date					
No	15 911 (53)	12 623 (58)	960 (54)	2328 (37)	<.001
Yes	13 966 (47)	9188 (42)	825 (46)	3953 (63)	

Abbreviations: CRC, colorectal cancer; DCRC, detected colorectal cancer; NA, not applicable; NSAID, nonsteroidal anti-inflammatory drug; PCCRC, postcolonoscopy colorectal cancer; VA, US Department of Veterans Affairs.

^a^ Data are presented as number (percentage) of patients unless otherwise indicated.

^b^ Pearson χ^2^ test; 2-sided Kruskal-Wallis rank sum test.

^c^ Other races include Asian (n = 119 [0.4%]), other (n = 357 [1%]), and unknown (n = 146 [0.5%]).

Compared with the DCRC group, patients with PCCRC-3y were more likely to have right-sided cancer (adjusted odds ratio [aOR], 2.01; 95% CI, 1.82-2.22; *P* < .001) and to have undergone colonoscopy outside the VA system (aOR, 2.58; 95% CI, 2.24-2.96; *P* < .001). In addition, PCCRC-3y was more likely than DCRC to present with bowel obstruction (aOR, 1.64; 95% CI, 1.29-2.07; *P* < .001) or peritonitis (aOR, 1.96; 95% CI, 1.06-3.37; *P* = .02) but not perforation (aOR, 1.66; 95% CI, 0.72-3.31; *P* = .19).

Mean (SE) follow-up in years after CRC diagnosis was 11.5 (0.05) for DCRC, 11.2 (0.2) for PCCRC-3y, and 8.0 (0.1) for no colonoscopy. The overall 5-year ACM rate was 47%, and the overall 5-year CSM rate was 29%. The 5-year ACM for PCCRC-3y and DCRC were comparable (46% vs 42%) as were the 5-year CSM rates (26% vs 25%). These rates were significantly lower than ACM (63%) and CSM (45%) in the no colonoscopy group. Kaplan-Meier survival curves for the 3 groups are depicted in the Figure. Univariate analyses are reported in eTable 1 in Supplement 1. Based on multivariable Cox proportional hazards analysis (Table 2), there was no significant difference in ACM and CSM between PCCRC-3y (aHR, 1.04; 95% CI, 0.98-1.11; *P* = .18) and DCRC (aHR, 1.04; 95% CI, 0.95-1.13; *P* = .42). However, compared with DCRC, patients with no colonoscopy had significantly higher ACM (aHR, 1.76; 95% CI, 1.70-1.82; *P* < .001) and CSM (aHR, 2.22; 95% CI, 2.12-2.32; *P* < .001). These results were similar when CRC stage was included in the model and when CRC anatomical location was removed from the model (eTables 2 and 3 in Supplement 1). For example, in the comparison between PCCRC-3y and DCRC with the inclusion of stage, the aHRs were 1.05 (95% CI, 0.99-1.11; *P* = .11) for ACM (eTable 2 in Supplement 1) and 1.05 (95% CI, 0.96-1.15; *P* = .29) for CSM (eTable 3 in Supplement 1).

**Figure.**
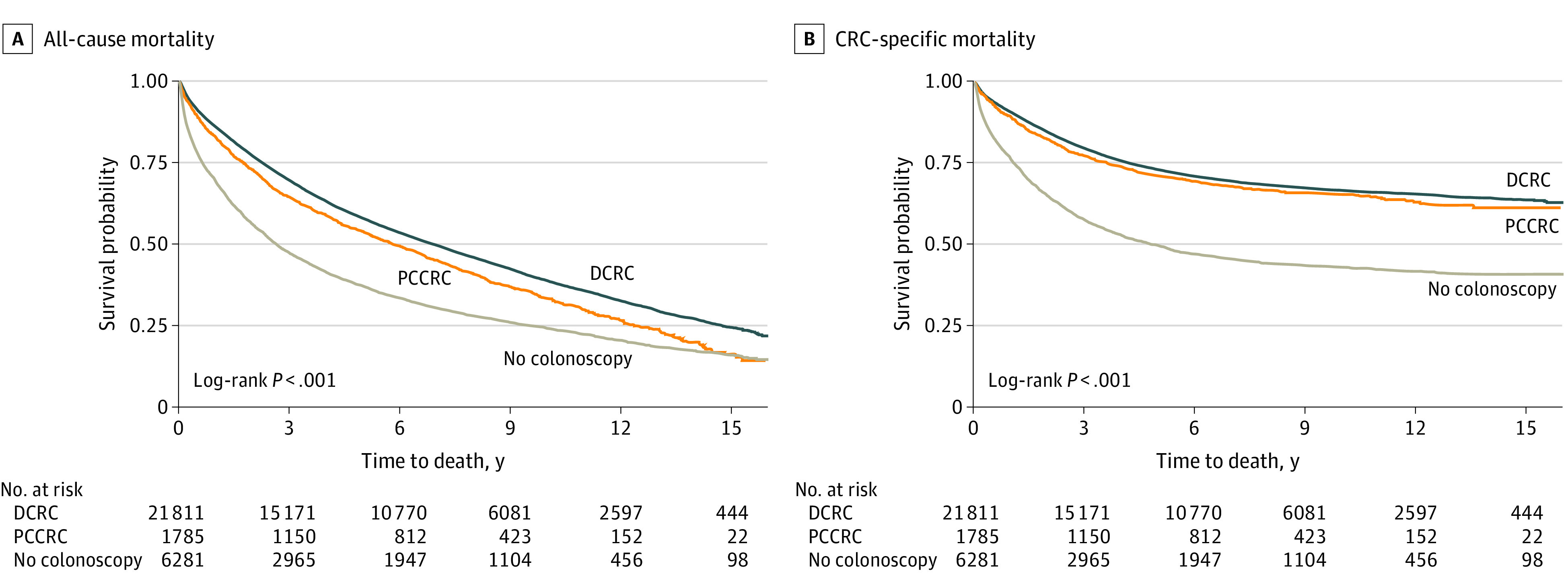
Kaplan-Meier Curves for All-Cause and Colorectal Cancer (CRC)–Specific Mortality DCRC indicates detected colorectal cancer; PCCRC, postcolonoscopy colorectal cancer.

**Table 2.** Multivariable Cox Proportional Hazards Regression Model of Time to All-Cause and CRC-Specific Death

Characteristic	All-cause death		CRC-specific death		
	aHR (95% CI)	*P* value	aHR (95% CI)	*P* value
Classification				
DCRC	1 [Reference]	NA	1 [Reference]	NA
PCCRC	1.04 (0.98-1.11)	.18	1.04 (0.95-1.13)	.42
No colonoscopy	1.76 (1.70-1.82)	<.001	2.22 (2.12-2.32)	<.001
Age	1.04 (1.04-1.04)	<.001	1.02 (1.01-1.02)	<.001
Sex				
Male	1 [Reference]	NA	1 [Reference]	NA
Female	0.83 (0.74-0.93)	.002	0.87 (0.74-1.02)	.082
Race				
White	1 [Reference]	NA	1 [Reference]	NA
Black	1.01 (0.97-1.05)	.60	1.09 (1.03-1.14)	.002
Other	0.97 (0.87-1.07)	.51	0.98 (0.85-1.13)	.748
Indicator for Hispanic ethnicity				
No	1 [Reference]	NA	1 [Reference]	NA
Yes	0.85 (0.80-0.90)	<.001	0.88 (0.81-0.95)	.002
Weighted Charlson Comorbidity Index score	1.17 (1.16-1.17)	<.001	1.16 (1.15-1.17)	<.001
Statin use				
No	1 [Reference]	NA	1 [Reference]	NA
Yes	0.88 (0.85-0.91)	<.001	0.80 (0.76-0.84)	<.001
NSAID use				
No	1 [Reference]	NA	1 [Reference]	NA
Yes	0.97 (0.93-1.02)	.20	0.98 (0.92-1.04)	.45
Aspirin use				
No	1 [Reference]	NA	1 [Reference]	NA
Yes	0.96 (0.93-0.99)	.02	0.89 (0.85-0.94)	<.001
Family history of CRC				
No	1 [Reference]	NA	1 [Reference]	NA
Yes	0.82 (0.73-0.92)	<.001	0.83 (0.70-0.98)	.02
Current smoker				
No	1 [Reference]	NA	1 [Reference]	NA
Yes	1.31 (1.28-1.35)	<.001	1.20 (1.15-1.25)	<.001
CRC location				
Left or rectum	1 [Reference]	NA	1 [Reference]	NA
Right	0.91 (0.88-0.93)	<.001	0.86 (0.82-0.90)	<.001
Both	1.20 (1.07-1.35)	.001	1.30 (1.12-1.52)	<.001
Undefined	1.43 (1.33-1.53)	<.001	1.56 (1.42-1.72)	<.001

Abbreviations: aHR, adjusted hazard ratio; CRC, colorectal cancer; DCRC, detected colorectal cancer; NA, not applicable; NSAID, nonsteroidal anti-inflammatory drug; PCCRC, postcolonoscopy colorectal cancer.

In a χ^2^ test of the association between endoscopist specialty (gastroenterology vs nongastroenterology) and group (PCCRC-3yr vs DCRC), we found that patients with PCCRC-3y had significantly lower odds of colonoscopy performance by a gastroenterology specialist compared with DCRC (odds ratio [OR], 0.48; 95% CI, 0.43-0.53; *P* < .001). However, multivariable Cox proportional hazards regression models within the PCCRC group showed that endoscopist specialty was not associated with ACM (aHR for gastroenterology vs nongastroenterology, 1.09; 95% CI, 0.97-1.22; *P* = .14) or CSM (aHR for gastroenterology vs nongastroenterology, 1.08; 95% CI, 0.90-1.28; *P* = .41). There was no association between location of performance of index colonoscopy (VA vs non-VA) and either ACM (aHR, 0.98; 95% CI, 0.92-1.04; *P* = .51) or CSM (aHR, 0.97; 95% CI, 0.89-1.07; *P* = .55).

In a secondary analysis, 3071 CRCs (10%) were classified as PCCRC-5y and 20 754 (70%) as DCRC. Similar to the primary analysis, there was no significant difference in ACM and CSM between PCCRC-5y (aHR, 1.01; 95% CI, 0.97-1.06; *P* = .58) and DCRC (aHR, 1.03; 95% CI, 0.96-1.11; *P* = .38).

## Discussion

In this cohort study that used VA-Medicare data to compare outcomes between veterans with PCCRC-3y, veterans with DCRC, and those with no prior or recent colonoscopy, we found a prevalence of PCCRC-3y of 6% among all patients with CRC and both ACM and CSM comparable with that of patients with detected CRC. We also found that compared with patients with DCRC, those with PCCRC-3y had comparable cancer stage distribution, higher predilection for proximal colon location, and increased risk of a medically urgent presentation (bowel obstruction or peritonitis). Performance of colonoscopy outside the VA was associated with PCCRC-3y but not with mortality.

Several studies^2,3,4,5,6,7,8,9,10,11,12,13,14^ published in non-VA settings have described PCCRC prevalence, clinical features, and risks factors and compared PCCRC characteristics with those of CRC detected at colonoscopy. A systematic review and meta-analysis^17^ reported a pooled rate of PCCRC-3y of 3.7%, or approximately 1 in 27 CRCs, and found that these cancers were 2.4 times as likely to arise in the proximal colon as the distal colon. This is similar to our findings, further corroborating the results of studies^18,19,20,21,22,23,24^ showing that colonoscopy affords lower levels of protection against right-sided colon cancer.

There are, however, challenges in extrapolating the findings of these previous studies^18,19,20,21,22,23,24^ to the current era because of marked heterogeneity in the study methods and definitions of PCCRC. The WEO has recently produced a consensus statement to help standardize nomenclature and study methods in the PCCRC field,^25^ notably recommending PCCRC-3y as a primary measure. Our study followed the WEO approach to the definition of PCCRC-3y . A recent meta-analysis^26^ based on the WEO guidelines reported a pooled prevalence of PCCRC-3y of 8.2% (95% CI, 6.9%-9.4%), comparable with our rate of 6%. The fact that our rate is at the lower end of the range reported in other health care settings should provide reassurance regarding the VA’s commitment to quality, particularly in a context where veterans can opt for non-VA care via the CHOICE (Veterans Access, Choice, and Accountability) and MISSION (Maintaining Internal Systems and Strengthening Integrated Outside Networks) acts.

Previous studies^7,11,12,13^ assessing the outcome of patients after PCCRC diagnosis have yielded inconsistent findings. The study by Singh et al^13^ in Manitoba and that of Erichsen et al^7^ in Denmark showed no difference in survival between DCRC and PCCRC. Conversely, Samadder et al^12^ found better survival and earlier-stage distribution for PCCRC compared with DCRC. Govindarajan et al^11^ published a population-based, retrospective cohort study of 45 104 patients diagnosed with CRC in Ontario, Canada, from 2003 to 2009, of whom 2804 (6.2%) had PCCRC diagnosed within 6 to 36 months of colonoscopy. Compared with detected CRC diagnosed within 6 months of colonoscopy, PCCRC was associated with a significantly higher likelihood of stage IV disease (17.2% vs 12.9%), lower 5-year survival (60.8% vs 68.3%, *P* < .001; aHR, 1.25; 95% CI, 1.17-1.32; *P* < .001), and lower likelihood of surgical resection (aOR, 0.61; 95% CI, 0.55-0.67; *P* < .001). Forsberg et al^9^ performed a population-based cohort study in Sweden; their PCCRC-3y rate was 7.2%, and patients with PCCRC had significantly shorter survival times than those with DCRC. Finally, a recent study by Yang et al^27^ assessed 4836 CRCs diagnosed in 3 large prospective cohorts and defined screen-detected and interval CRCs based on the timing of diagnosis with regard to the baseline screening procedure and the next recommended surveillance examination. They found that interval CRCs were associated with higher ACM and CSM than screen-detected CRC.

In our study, we found that patients with DCRC and PCCRC had comparable all-cause and CRC-specific mortality. We intentionally chose mortality instead of survival time as the outcome of interest to mitigate the risk of lead time and length of time biases that are more likely to impact studies assessing survival rates after cancer diagnosis. We assessed CSM because it is considered the most robust cancer outcome measure and to address confounding caused by comorbidity burden and the healthy-user bias that could limit studies reporting ACM alone. Although most PCCRC cases are believed to reflect a failure at the time of the index colonoscopy (missed cancer or precursor lesion or incomplete polypectomy), the most likely scenario explaining the lack of impact on mortality is that most of these cancers are still small and at an early stage within a 3-year interval after colonoscopy. The observation that patients with DCRC and patients with PCCRC-3y had similar cancer stage distribution supports this notion and is consistent with a dwell time for PCCRC-3y similar to that of sporadic DCRC. In turn, this finding supports the notion that accelerated neoplasia progression may not be a major factor in the pathogenesis of most PCCRC. In addition, our findings were robust and unchanged regardless of adjustment for CRC stage or anatomic location. Molecular analyses were beyond the scope of this work and may have yielded additional insights regarding inherent biological differences between PCCRC and DCRC. However, in the recent study by Yang et al,^27^ there were no significant mutational differences between screen-detected and interval CRCs, and the mortality differences between the 2 groups appear to be driven by higher proportions of stage IV disease among interval CRC cases. These observations, together with the relatively high overall rate of interval CRC reported in this study (approximately 15%), suggest that colonoscopy quality considerations are more pertinent with respect to the pathogenesis of PCCRC-3y, rather than inherent molecular differences.

We found that patients with CRC and no prior colonoscopy had significantly worse ACM and CSM than either PCCRC-3y or DCRC. This finding is likely driven by the fact that nearly 40% of patients in this group had stage IV cancers at diagnosis, compared with 15% to 17% in the other 2 groups. This observation is not surprising because patients who recently underwent colonoscopy are expected to benefit from the detection of cancer in its early stages and from CRC prevention via polypectomy. Of note, nearly 93% of patients in the no colonoscopy group consisted of individuals who underwent colonoscopy more than 10 years before CRC diagnosis or had no colonoscopy that could be identified in the Corporate Data Warehouse or CMS data. This finding underscores the importance of regular and programmatic CRC screening and of measuring colonoscopy quality. Our methods allowed capturing exposure to colonoscopy back to 1997; however, this timeline encompassed an era when procedure quality and its impact on CRC outcomes were not well appreciated. Overall, the finding of cancer downstaging and decreased mortality among patients exposed to colonoscopy broadly reaffirms the benefits of CRC screening.

Previous studies^28,29,30,31^ have shown that endoscopist specialty is a factor associated with colonoscopy quality, with gastroenterologists as a group outperforming practitioners from other specialties. In our study, we found lower odds of PCCRC-3y when procedures were performed by gastroenterologists. This observation highlights the critical importance of universal commitment to high-quality colonoscopy and programmatic measurement and monitoring of quality parameters, as is currently standard within the VHA and most health care systems in the US.

### Limitations

Despite the strengths of a large sample size and rigorous approach to CRC classification and analysis of outcomes, our study has limitations. Mortality data were available only through December 31, 2018, which limited our ability to assess PCCRC-3y and DCRC in a more contemporary context, particularly with regard to current colonoscopy quality paradigms. Notably, we were unable to measure colonoscopy quality metrics, such as adenoma detection or cecal intubation rates. We were able to account for colonoscopies that were performed outside the VA and paid for by the VA, but it is possible that some procedures (eg, paid for by a patient and not documented in the medical record) were missed by our approach, leading to potential misclassification of some cases. Although the specifics of our veteran patient population (mostly male, older, and White) could be viewed as limiting generalizability, our PCCRC prevalence estimates are consistent with those of numerous previous studies^11,26^ in other settings in which both sexes are well represented, which supports the validity of our findings.

## Conclusions

In this large VA-Medicare study, we found that PCCRC-3y constitutes 6% of CRCs. Compared with CRC detected by colonoscopy, PCCRC-3y has comparable ACM and CSM. Additional study of the factors associated with PCCRC and the interventions that effectively lower its occurrence is warranted.

## Notes

Supplement 1. **eMethods.** Data Sources **eTable 1.** Hazard Ratios and 95% Confidence Intervals From Univariable Cox Proportional Regression Modeling of ACM and CSM **eTable 2.** Hazard Ratios and 95% Confidence Intervals From Multivariable Cox Proportional Regression Modeling of ACM, With Stage and Without Anatomic Location **eTable 3.** Hazard Ratios and 95% Confidence Intervals From Multivariable Cox Proportional Regression Modeling of CSM, With Stage and Without Anatomic Location

Supplement 2.
Data Sharing Statement
