# Supplementary material for: Mortality After Postcolonoscopy Colorectal Cancer in the Veterans Affairs Health Care System
Source: JAMA Netw Open. 2023 Apr 6;6(4):e236693. doi: 10.1001/jamanetworkopen.2023.6693 (PMC10080371; doi:10.1001/jamanetworkopen.2023.6693)
Supplement: Supplement 1. — eMethods. Data Sources eTable 1. Hazard Ratios and 95% Confidence Intervals From Univariable Cox Proportional Regression Modeling of ACM and CSM eTable 2. Hazard Ratios and 95% Confidence Intervals From Multivariable Cox Proportional Regression Modeling of ACM, With Stage and Without Anatomic Location eTable 3. Hazard Ratios and 95% Confidence Intervals From Multivariable Cox Proportional Regression Modeling of CSM, With Stage and Without Anatomic Location [file jamanetwopen-e236693-s001.pdf]

Supplementary Online Content

Kahi CJ, Myers LJ, Monahan PO, Barker BC, Stump TE, Imperiale TF. Mortality after postcolonoscopy colorectal cancer in the Veterans Affairs health care system. *JAMA Netw Open*. 2023;6(4):e236693. doi:10.1001/jamanetworkopen.2023.6693

eMethods. Data Sources

eTable 1. Hazard Ratios and 95% Confidence Intervals From Univariable Cox Proportional Regression Modeling of ACM and CSM

eTable 2. Hazard Ratios and 95% Confidence Intervals From Multivariable Cox Proportional Regression Modeling of ACM, With Stage and Without Anatomic Location

eTable 3. Hazard Ratios and 95% Confidence Intervals From Multivariable Cox Proportional Regression Modeling of CSM, With Stage and Without Anatomic Location

This supplementary material has been provided by the authors to give readers additional information about their work.

© 2023 Kahi CJ et al. *JAMA Network Open.*

eMethods. Data Sources

1. Corporate Data Warehouse (CDW) is a national repository of VHA clinical and administrative systems. CDW data are stored in relational databases. Data from multiple domains, such as oncology, inpatient, outpatient, laboratory, vital signs, orders, health factors, and pharmacy data, are available. Purchased care data (previously known as fee-basis data) are also available. Textual information utilities (TIU) documents are available within CDW; TIU files store textual information from CPRS/VISTA, such as progress notes and admission and discharge summaries. The oncology tables in CDW include the same information that is available in the VA Central Cancer Registry (VACCR), with the benefit of being more up-to-date than VACCR. Information in the oncology tables includes patient demographics, date of diagnosis, pathologic confirmation, cancer location, cancer stage, first and subsequent courses of treatment, family history, and outcomes. Cancer site is identified using ICD for Oncology (ICD-O-3).

2. VA-Medicare merged data: The VA-Medicare Data Merge Initiative aims to provide access to Medicare data for VA researchers. VA-Medicare data were used to identify colonoscopies, medical history, and other measures of healthcare utilization.

3. VA Vital Status File: The VA-VSF is a registry containing death dates for veterans from all available sources (BIRLS, PTF, Medicare, and SSA). Using the National Death Index (NDI) as the gold standard, the VA-VSF has a sensitivity of 98.3%, specificity of 99.8%, and 97.6% exact agreement with dates of death from the NDI.

4. VA/Department of Defense (DoD) Mortality Data Repository (MDR): Given that the VA-VSF does not contain information regarding cause of death, we supplemented the VA-VSF with data from the VA/DoD MDR, which contains information from the National Death Index (NDI), including primary and contributing cause of death using ICD-10 codes. Data from the VA/DoD MDR were available through 12/31/2018.

© 2023 Kahi CJ et al. *JAMA Network Open.*

eTable 1. Hazard Ratios and 95% Confidence Intervals From Univariable Cox Proportional Regression Modeling of ACM and CSM

		ACM			CSM		
Characteristic	N	HR	95% CI	p-value	HR	95% CI	p-value
**Classification**	29,877			**<0.001**			**<0.001**
DCRC		—	—		—	—	
PCCRC-3y		1.18	1.11, 1.25	**<0.001**	1.08	0.99, 1.19	**0.074**
No Colonoscopy		1.69	1.63, 1.74	**<0.001**	2.25	2.15, 2.34	**<0.001**
**Age**	29,877	1.04	1.03, 1.04	**<0.001**	1.01	1.01, 1.01	**<0.001**
**Sex**	29,877			**<0.001**			**0.002**
Male		—	—		—	—	
Female		0.72	0.64, 0.81	**<0.001**	0.78	0.67, 0.92	**0.003**
**Race**	29,877			0.274			**<0.001**
White		—	—		—	—	
Black		1.03	0.99, 1.07	**0.149**	1.16	1.11, 1.22	**<0.001**
Other		0.97	0.88, 1.07	0.533	0.99	0.86, 1.14	0.935
**Indicator for Hispanic ethnicity**	29,877			**<0.001**			**0.041**
No		—	—		—	—	
Yes		0.88	0.83, 0.93	**<0.001**	0.92	0.85, 1.00	**0.044**
**Weighted Charlson Score**	29,876	1.17	1.17, 1.18	**<0.001**	1.16	1.15, 1.17	**<0.001**
**Statin use**	29,876			0.323			**<0.001**
No		—	—		—	—	
Yes		1.01	0.99, 1.04	0.323	0.82	0.79, 0.86	**<0.001**
**NSAID use**	29,876			**<0.001**			**<0.001**
No		—	—		—	—	
Yes		0.89	0.85, 0.93	**<0.001**	0.89	0.83, 0.95	**<0.001**
**Aspirin use**	29,876			**<0.001**			**<0.001**
No		—	—		—	—	
Yes		1.09	1.05, 1.12	**<0.001**	0.89	0.85, 0.94	**<0.001**
**Family history of CRC**	29,876			**<0.001**			**<0.001**
No		—	—		—	—	
Yes		0.72	0.64, 0.80	**<0.001**	0.72	0.62, 0.85	**<0.001**
**Current smoker**	29,876			**<0.001**			**<0.001**
No		—	—		—	—	
Yes		1.15	1.12, 1.18	**<0.001**	1.18	1.13, 1.23	**<0.001**
**CRC location**	29,877			**<0.001**			**<0.001**
Left or Rectum		—	—		—	—	
Right		1.07	1.04, 1.10	**<0.001**	0.94	0.90, 0.98	**0.002**
Both		1.24	1.11, 1.39	**<0.001**	1.30	1.12, 1.51	**<0.001**
Undefined		1.66	1.55, 1.79	**<0.001**	1.84	1.68, 2.03	**<0.001**
**Cancer stage**	29,877			**<0.001**			**<0.001**
I		—	—		—	—	
II		1.47	1.41, 1.53	**<0.001**	2.86	2.64, 3.10	**<0.001**
III		1.69	1.63, 1.77	**<0.001**	4.76	4.40, 5.14	**<0.001**
IV		7.74	7.43, 8.06	**<0.001**	28.1	26.1, 30.3	**<0.001**

© 2023 Kahi CJ et al. *JAMA Network Open.*

eTable 2. Hazard Ratios and 95% Confidence Intervals From Multivariable Cox Proportional Regression Modeling of ACM, With Stage and Without Anatomic Location

	Multivariable with Stage			Multivariable without Location		
Characteristic	HR[1]	95% CI[1]	p-value	HR[1]	95% CI[1]	p-value
**Classification**			**<0.001**			**<0.001**
DCRC	—	—		—	—	
PCCRC-3y	1.05	0.99, 1.11	0.109	1.03	0.97, 1.09	0.323
No Colonoscopy	1.32	1.27, 1.36	**<0.001**	1.78	1.72, 1.84	**<0.001**
**Age**	1.04	1.04, 1.05	**<0.001**	1.04	1.04, 1.04	**<0.001**
**Sex**			**0.001**			**<0.001**
Male	—	—		—	—	
Female	0.82	0.73, 0.93	**0.002**	0.82	0.72, 0.92	**<0.001**
**Race**			0.654			0.717
White	—	—		—	—	
Black	0.98	0.95, 1.02	0.390	1.01	0.97, 1.05	0.585
Other	0.98	0.89, 1.08	0.701	0.97	0.88, 1.07	0.571
**Indicator for Hispanic ethnicity**			**<0.001**			**<0.001**
No	—	—		—	—	
Yes	0.83	0.78, 0.88	**<0.001**	0.86	0.81, 0.91	**<0.001**
**Weighted Charlson Score**	1.12	1.12, 1.13	**<0.001**	1.16	1.16, 1.17	**<0.001**
**Statin use**			**<0.001**			**<0.001**
No	—	—		—	—	
Yes	0.93	0.90, 0.96	**<0.001**	0.88	0.85, 0.91	**<0.001**
**NSAID use**			0.381			0.211
No	—	—		—	—	
Yes	0.98	0.94, 1.03	0.382	0.97	0.93, 1.02	0.212
**Aspirin use**			0.356			**0.013**
No	—	—		—	—	
Yes	0.98	0.95, 1.02	0.357	0.96	0.92, 0.99	**0.013**
**Family history of CRC**			**0.013**			**<0.001**
No	—	—		—	—	
Yes	0.87	0.77, 0.97	**0.015**	0.82	0.73, 0.92	**<0.001**
**Current smoker**			**<0.001**			**<0.001**
No	—	—		—	—	
Yes	1.28	1.24, 1.32	**<0.001**	1.32	1.28, 1.35	**<0.001**
**CRC location**			**<0.001**			
Left or Rectum	—	—		NA	NA	NA
Right	0.91	0.88, 0.94	**<0.001**			
Both	1.08	0.96, 1.21	0.197			
Undefined	1.22	1.14, 1.32	**<0.001**			
**Cancer stage**			**<0.001**			
I	—	—		NA	NA	NA
II	1.34	1.28, 1.39	**<0.001**			
III	1.61	1.55, 1.68	**<0.001**			
IV	6.91	6.62, 7.21	**<0.001**			

© 2023 Kahi CJ et al. *JAMA Network Open.*

eTable 3. Hazard Ratios and 95% Confidence Intervals From Multivariable Cox Proportional Regression Modeling of CSM, With Stage and Without Anatomic Location

	Multivariable with Stage			Multivariable without Location		
Characteristic	HR[1]	95% CI[1]	p-value	HR[1]	95% CI[1]	p-value
**Classification**			**<0.001**			**<0.001**
DCRC	—	—		—	—	
PCCRC-3y	1.05	0.96, 1.15	0.290	1.02	0.93, 1.11	0.682
No Colonoscopy	1.36	1.30, 1.43	**<0.001**	2.25	2.15, 2.35	**<0.001**
**Age**	1.03	1.02, 1.03	**<0.001**	1.01	1.01, 1.02	**<0.001**
**Sex**			**0.047**			**0.040**
Male	—	—		—	—	
Female	0.85	0.72, 1.00	0.053	0.85	0.72, 1.00	**0.046**
**Race**			0.415			**0.007**
White	—	—		—	—	
Black	1.03	0.98, 1.09	0.193	1.08	1.03, 1.14	**0.002**
Other	0.99	0.86, 1.14	0.849	0.98	0.85, 1.13	0.797
**Indicator for Hispanic ethnicity**			**<0.001**			**0.006**
No	—	—		—	—	
Yes	0.85	0.78, 0.92	**<0.001**	0.89	0.82, 0.97	**0.007**
**Weighted Charlson Score**	1.09	1.09, 1.10	**<0.001**	1.16	1.15, 1.17	**<0.001**
**Statin use**			**<0.001**			**<0.001**
No	—	—		—	—	
Yes	0.88	0.84, 0.93	**<0.001**	0.80	0.76, 0.83	**<0.001**
**NSAID use**			0.995			0.474
No	—	—		—	—	
Yes	1.00	0.94, 1.07	0.995	0.98	0.92, 1.04	0.476
**Aspirin use**			**0.006**			**<0.001**
No	—	—		—	—	
Yes	0.93	0.88, 0.98	**0.007**	0.89	0.84, 0.93	**<0.001**
**Family history of CRC**			0.426			**0.021**
No	—	—		—	—	
Yes	0.94	0.79, 1.10	0.431	0.83	0.70, 0.98	**0.025**
**Current smoker**			**<0.001**			**<0.001**
No	—	—		—	—	
Yes	1.13	1.09, 1.18	**<0.001**	1.20	1.15, 1.25	**<0.001**
**CRC location**			**<0.001**	NA	NA	NA
Left or Rectum	—	—				
Right	0.87	0.83, 0.91	**<0.001**			
Both	1.06	0.91, 1.23	0.473			
Undefined	1.21	1.10, 1.33	**<0.001**			
**Cancer stage**			**<0.001**	NA	NA	NA
I	—	—				
II	2.68	2.47, 2.91	**<0.001**			
III	4.54	4.20, 4.90	**<0.001**			
IV	24.7	22.9, 26.6	**<0.001**			

© 2023 Kahi CJ et al. *JAMA Network Open.*
