# Supplementary material for: Mortality After Postcolonoscopy Colorectal Cancer in the Veterans Affairs Health Care System
Source: JAMA Netw Open. 2023 Apr 6;6(4):e236693. doi: 10.1001/jamanetworkopen.2023.6693 (PMC10080371; doi:10.1001/jamanetworkopen.2023.6693)
Supplement: Supplement 2. — Data Sharing Statement [file jamanetwopen-e236693-s002.pdf]

Data Sharing Statement

Kahi. Mortality After Postcolonoscopy Colorectal Cancer in the Veterans Affairs Health Care System. *JAMA Netw Open*. Published April 06, 2023.
doi:10.1001/jamanetworkopen.2023.6693

Data
Data available: No
